# The complete chloroplast genome of *Swertia tetraptera* and phylogenetic analysis

**DOI:** 10.1080/23802359.2019.1698368

**Published:** 2019-12-12

**Authors:** Lucun Yang, Feng Xiong, Yuanming Xiao, Jingjing Li, Cheng Chen, Guoying Zhou

**Affiliations:** a Qinghai Key Laboratory of Qinghai-Tibet Plateau Biological Resources, Northwest Institute of Plateau Biology, Chinese Academy of Sciences, Xining, China;; b University of Chinese Academy of Sciences, Beijing, China;; c Qinghai Normal University, Xining, China

**Keywords:** *Swertia tetraptera*, chloroplast genome, phylogenetic position

## Abstract

*Swertia tetraptera*, native to the Qinghai-Tibetan Plateau, is an important traditional Chinese medicine. Although researchers have done a lot of work on it, the phylogenetic position of *S. tetraptera* within Swertia has still not been solved. Chloroplast genome sequences play a significant role in the development of molecular markers in plant phylogenetic and population genetic studies. In present study, we determined the complete chloroplast genome sequences for *S. tetraptera* using IIumina sequencing. The total length of the complete chloroplast genome of *S. tetraptera* is 152,840 bp, of which the GC content is 37.95%. The genome encodes 130 functional genes, including 85 protein-coding genes, 37 tRNA, and 8 rRNA. Phylogenetic analysis suggested that *S. tetraptera* forms monophyletic group with *Halenia corniculata* which shows closed relationship with the *Halenia*.

*Swertia tetraptera* Maxim, belonging to Gentianaceae family, Gentianales order, Asteridae subclass, is an alpine annual herbaceous plant endemic to the Qinghai-Tibetan Plateau (QTP). It is mainly distributed in Qinghai, Gansu, Sichuan and Xizang Provinces, occurring primarily in moist hillsides and shrub locations with an elevation of 2500–4700 m. As an important traditional Chinese medicine, researchers mainly focused on its chemical composition (Zhao et al. 2016; Li et al. 2017). However, the phylogenetic position of *S. tetraptera* within Swertia has still not been solved. Different researchers have different views on the phylogenetic position of *S. tetraptera* based on the various methods (Grisebach 1839; He and Liu 1980; Yuan and Küpfer 1995; Xue et al. 1999; Liu et al. 2001; Chassot et al. 2001; Chassot and Von Hagen 2008; He et al. 2013). Therefore, it is necessary to use a new method to solve the phylogenetic position of *S. tetraptera*. Compared with the nuclear genome, the chloroplast genome is small, and the rate of nucleotide substitutions is so low that the chloroplast genome is considered to be an ideal system for studies on phylogeny (Wei et al. 2005). To data, there are only 17 complete chloroplast genomes of Gentianaceae on the NCBI public database. The complete chloroplast genome of *S. tetraptera* has not been reported. Here, we confirmed the complete chloroplast genome of *S. tetraptera* and constructed phylogenetic trees to provide insight into phylogenetic relationships of *S. tetraptera* and related species.

In present study, a wild individual of *S. tetraptera* was sampled from Arou village, Qilian country in Qinghai province of China (100°27.017′E, 38°04.315′N, 3084 m). A voucher specimen was deposited in the HNWP with voucher number of QHGC20190820. Genomic DNA of single individual was extracted from fresh leaves following the improved CTAB protocol (Doyle 1991). After DNA sample was fragmented, an Illumina pair-endlibrary was constructed and sequenced by the Illumina HiSeq 4000 platform. And then, the complete chloroplast genome was assembled and annotated with the SPAdes (Bankevich et al. 2012) and DOGMA (Wyman et al. 2004), respectively. The annotated genomic sequence had been submitted to GenBank with the accession number SAMN13258262.

The total length of the complete chloroplast genome of *S. tetraptera* is 152,840 bp, of which the GC content is 37.95%. A large single copy (LSC: 83,177 bp), a small single copy (SSC: 18,305 bp) and two inverted repeat (IR: 25,679 bp) regions make up the typical quadripartite structure of the chloroplast genome of *S. tetraptera*. The genome encodes 130 functional genes, including 85 protein-coding genes, 37 tRNA, and 8 rRNA. A total of 18 genes were duplicated in the IR regions including seven tRNA, four rRNA, and seven protein-coding genes. The genome organization, gene/intron content and gene relative positions of the newly sequenced plastid genome were almost identical to other Gentianaceae species.

We used the complete chloroplast genomes of *S. tetraptera* and 19 other species from Gentianaceae to construct the Phylogenetic tree. And *Carissa macrocarpa* (Apocynaceae) was used as an outgroup. Maximum-likelihood (ML) analysis demonstrated that *S. tetraptera* formed a clade with *Halenia corniculata* with high bootstrap values (Figure 1). And then, it clustered a clade branch with the other species in Swertia. The newly characterized *S. tetraptera* chloroplast genome provided a new insight for the phylogenetic position of *S. tetraptera*.

**Figure 1.**
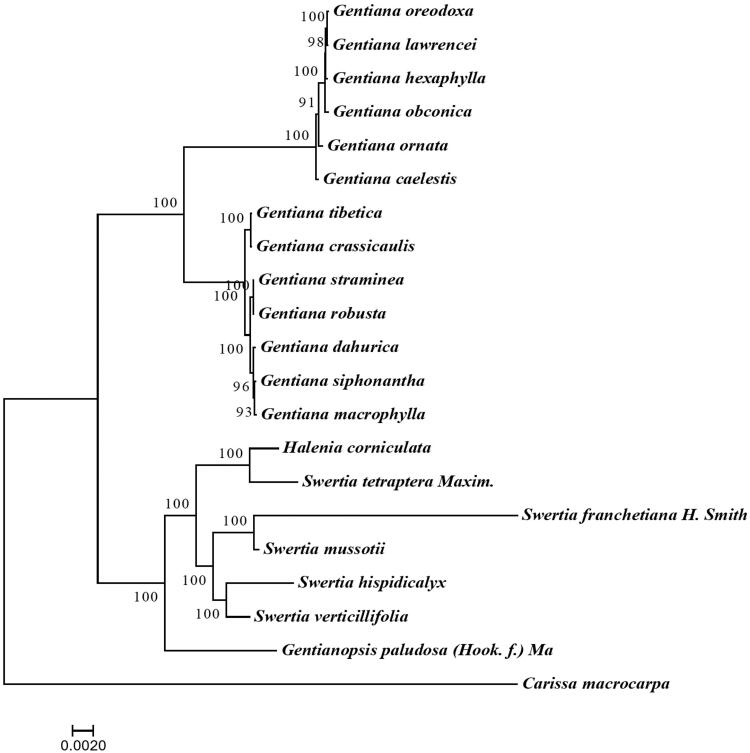
Maximum likelihood phylogenetic tree based on 21 complete chloroplast genome sequences. The number on each node indicates the bootstrap value. Accession numbers: *Gentiana oreodoxa* NC_037982; *Gentiana lawrencei* KX096882; *Gentiana hexaphylla* NC_037980; *Gentiana obconica* NC_037981; *Gentiana ornate* MG192308; *Gentiana caelestis* NC_037979; *Gentiana tibetica* NC_030319; *Gentiana crassicaulis* KY595463; *Gentiana straminea* KJ657732; *Gentiana robusta* KT159969; *Gentiana dahurica* NC_039572; *Gentiana siphonantha* NC_039573; *Gentiana macrophylla* NC_035719; *Halenia corniculata* NC_042674; *Swertia mussotii* NC_031155; *Swertia hispidicalyx* NC_044474; *Swertia verticillifolia* MF795137; *Carissa macrocarpa* KX364402.
